# Plasma-Assisted Synthesis of Multicomponent Nanoparticles Containing Carbon, Tungsten Carbide and Silver as Multifunctional Filler for Polylactic Acid Composite Films

**DOI:** 10.3390/polym13070991

**Published:** 2021-03-24

**Authors:** Nichapat Boonyeun, Ratana Rujiravanit, Nagahiro Saito

**Affiliations:** 1 The Petroleum and Petrochemical College, Chulalongkorn University, Bangkok 10330, Thailand; Nichapat.B@student.chula.ac.th; 2 Center of Excellence on Petrochemical and Materials Technology, Chulalongkorn University, Bangkok 10330, Thailand; 3 Department of Chemical Systems Engineering, Graduate School of Engineering, Nagoya University, Nagoya 464-8603, Japan; Hiro@sp.material.nagoya-u.ac.jp

**Keywords:** atmospheric pressure plasma, filler, polylactic acid, polymer composite

## Abstract

Multicomponent nanoparticles containing carbon, tungsten carbide and silver (carbon-WC-Ag nanoparticles) were simply synthesized via in-liquid electrical discharge plasma, the so-called solution plasma process, by using tungsten electrodes immersed in palm oil containing droplets of AgNO_3_ solution as carbon and silver precursors, respectively. The atomic ratio of carbon:W:Ag in carbon-WC-Ag nanoparticles was 20:1:3. FE-SEM images revealed that the synthesized carbon-WC-Ag nanoparticles with particle sizes in the range of 20–400 nm had a spherical shape with a bumpy surface. TEM images of carbon-WC-Ag nanoparticles showed that tungsten carbide nanoparticles (WCNPs) and silver nanoparticles (AgNPs) with average particle sizes of 3.46 nm and 72.74 nm, respectively, were dispersed in amorphous carbon. The carbon-WC-Ag nanoparticles were used as multifunctional fillers for the preparation of polylactic acid (PLA) composite films, i.e., PLA/carbon-WC-Ag, by solution casting. Interestingly, the coexistence of WCNPs and AgNPs in carbon-WC-Ag nanoparticles provided a benefit for the co-nucleation ability of WCNPs and AgNPs, resulting in enhanced crystallization of PLA, as evidenced by the reduction in the cold crystallization temperature of PLA. At the low content of 1.23 wt% carbon-WC-Ag nanoparticles, the Young’s modulus and tensile strength of PLA/carbon-WC-Ag composite films were increased to 25.12% and 46.08%, respectively. Moreover, the PLA/carbon-WC-Ag composite films possessed antibacterial activities.

## 1. Introduction

Nowadays, due to increasing environmental concerns and climate change worldwide, bio-derived and biodegradable polymers such as polylactic acid (PLA), polyhydroxybutyrate (PHB) and poly(3-hydroxybutyrate-co-3-hydroxyvalerate) (PHBV) have received much attention as alternatives to petroleum-based polymers [1,2,3]. Among bio-derived and biodegradable polymers, PLA is one of the most studied polymers and has been investigated for many applications, such as packaging materials [4], membranes for separation processes [5] and biomaterials for biomedical applications [6]. Nevertheless, the major drawbacks of PLA that limit its utilization as a replacement for petroleum-based polymers are low thermal stability, inferior mechanical properties and low crystallization rate when compared with petroleum-based polymers. Therefore, the incorporation of fillers into PLA to obtain PLA composites has been considered as an effective method to overcome these limitations. To date, PLA composites have been developed by the incorporation of a wide variety of fillers, such as clay [7], silver nanoparticles [8], zinc oxide (ZnO) [9], tungsten disulfide (WS_2_) nanotubes [10] and various types of carbon materials [11,12,13]. Among the investigated fillers, carbon materials have been considered as efficient candidates for the development of PLA composites because of their outstanding properties, such as high surface area, high thermal and chemical stability, high absorption ability and low cost [14,15]. However, the incorporation of monocomponent fillers could improve only specific properties of polymers [16,17]. Hence, the incorporation of multicomponent fillers into polymers has been employed in order to accomplish simultaneous improvement of the multifunctional properties of polymers, such as mechanical properties, thermal properties, crystallization behavior and antibacterial activities [18,19]. According to previous reports, the aggregation and non-homogeneous dispersion of each component in polymer matrices could occur, resulting in the deterioration of the properties of polymers [20,21]. To overcome these problems, nanohybrid fillers such as cellulose/silver nanoparticles and multiwall carbon nanotubes (MWCNTs)/silver nanoparticles were fabricated and incorporated into polymers [8,22,23]. However, the preparation methods of the nanohybrid fillers commonly involved time-consuming processes and the utilization of chemical agents.

The solution plasma process (SPP), an electrical discharge of plasma occurring in a liquid phase, has been considered as an emerging technology that has been investigated in carbon synthesis [24,25,26], surface modification of carbon materials [27], synthesis of metal nanoparticles [28,29] and synthesis of carbon-supported metal nanoparticles [30,31]. SPP is operated by applying electric potential between a pair of metal electrodes that are immersed in a liquid phase. Energetic electrons that are released from the electrodes collide with molecules near the electrodes, resulting in the formation of a variety of highly active species, such as excited molecules, free electrons, free radicals and positively and negatively charged species. Moreover, as a result of electron bombardment at the surfaces of electrodes, the erosion of the electrodes may occur, leading to the formation of metal nanoparticles released from the electrodes [30,32]. For example, when tungsten electrodes were used in carbon synthesis by SPP, to some extent, tungsten carbide nanoparticles were generated together with the formation of carbon as a result of the sputtering of tungsten electrodes [33]. Under this circumstance, SPP can provide several benefits, such as accelerating reaction rate, lowering reaction temperature and reduction of chemicals used in various reactions, e.g., synthesis of metal nanoparticles [34,35]. Recently, different types of organic solvents, including benzene, cyclohexane and hexane, have been investigated as carbon precursors to synthesize carbon by using SPP [24,33,36]. However, the organic solvents are high-cost raw materials and their wastes from the reactions may cause environmental problems. As an alternative to organic solvents, palm oil, a carbon-rich renewable raw material consisting of triglycerides and fatty acids [37,38], is a potential carbon precursor for synthesis of carbon by SPP. It was postulated that long-chain hydrocarbon moieties in fatty acids that are present in palm oil can be converted to carbon by SPP. The mechanism for the conversion of carbon precursors to carbon materials by SPP was proposed by Morishita T et al., 2016 [36].

In the last decade, inorganic fillers with intrinsic antimicrobial properties, such as titanium dioxide [39,40], zinc oxide [41], graphene oxide (GO) [42], metal-organic frameworks (MOFs) [43] and silver nanoparticles (AgNPs) [44], have been applied to polymer composites in order to introduce antimicrobial functions to the composites. It is known that AgNPs are one of the most powerful antimicrobial agents. Nowadays, AgNPs are extensively utilized in a broad range of applications, such as conductive materials [45], catalysts [46], anticancer [47] and biomaterials [48]. However, the stability of AgNPs is an important issue hindering their application. As their particle sizes are in the nano-scale, the surface energy of AgNPs is high, resulting in a tendency to undergo aggregation [49]. The aggregation of AgNPs results in a reduction in their performance, such as their catalytic efficiency and antimicrobial activities [50,51]. In order to prevent the aggregation of AgNPs, the deposition of AgNPs on various supporting materials, such as silica, zeolite and carbon, has been explored [52,53]. Among the most widely used supporting materials, carbon materials have many advantages, including high surface area, stability in acid and base media and low cost. Therefore, carbon materials are considered as potential candidates for use as supporting materials for AgNPs to obtain carbon-supported AgNPs [54,55]. Nowadays, carbon-supported AgNPs are extensively used as reinforcing fillers for polymer composites [22,56,57]. According to the literature, carbon-supported AgNPs can be synthesized by several methods, such as hydrothermal reduction [58], thermal annealing [56] and sputtering deposition [59]. However, the preparation of carbon-supported AgNPs is generally based on multi-step processes together with the use of reducing agents and long reaction times.

In this study, multicomponent nanoparticles containing carbon, tungsten carbide and silver, i.e., carbon-WC-Ag nanoparticles, were synthesized in one step via SPP by using palm oil containing droplets of silver nitrate (AgNO_3_) solution as carbon and silver precursors, respectively. By applying SPP, carbon and AgNPs were synthesized from palm oil and AgNO_3_, respectively, whereas tungsten carbide nanoparticles (WCNPs) were generated by the sputtering of tungsten electrodes together with the simultaneous formation of carbon during plasma discharge. The morphology and elemental composition of carbon-WC-Ag nanoparticles were characterized. Carbon-WC-Ag nanoparticles were then used as multifunctional fillers for the preparation of polylactic acid (PLA) composite films, i.e., PLA/carbon-WC-Ag, by solution casting. The effect of carbon-WC-Ag nanoparticles on the non-isothermal crystallization behavior and mechanical and thermal properties of the obtained PLA composite films was investigated. The antibacterial activities of PLA/carbon-WC-Ag composite films against *Escherichia coli*, a Gram-negative bacterium, and *Staphylococcus aureus*, a Gram-positive bacterium, were also examined.

## 2. Materials and Methods

### 2.1. Materials

Polylactic acid (PLA) (Ingeo^TM^ 4043D) with 94% L-lactide and 6% D-lactide was provided by Natureworks LLC (Minnetonka, MN, USA) (molecular weight (M_w_) of 1.5 × 10^5^ g/mol and polydispersity index (PDI) of 1.81). The melt flow index and density of PLA are 6.0 g/10 min and 1.24 g/cm^3^, respectively. Activated charcoal was purchased from Sigma-Aldrich Inc. (Lyon, France). Silver nitrate (AgNO_3_) was supplied by Fisher Scientific Co., Ltd. (Loughboroug, UK). Tungsten (W) and silver (Ag) electrodes (diameter of 1 mm, 99.95% purity) were obtained from The Nilaco Corporation, Japan. Chloroform (CHCl_3_) and hexane (C_6_H_8_) were analytical-grade and bought from RCI Labscan Co., Ltd. (Bangkok, Thailand). Palm oil was provided by Oleen Co., Ltd. (Samutsakhon, Thailand). *Escherichia coli* strain TISTR 527 and *Stapphylococcus aureus* strain TISTR 2329 were supplied by the Microbiological Resource Center, Thailand Institute of Scientific and Technological Research (TISTR). Bacteriological-grade beef extract, peptone and agar powder were purchased from HiMedia Laboratory Co., Ltd. (Maharashtra, India).

### 2.2. Preparation of Carbon-WC, Carbon-Ag and Carbon-WC-Ag Nanoparticles via SPP

**Figure 1:**
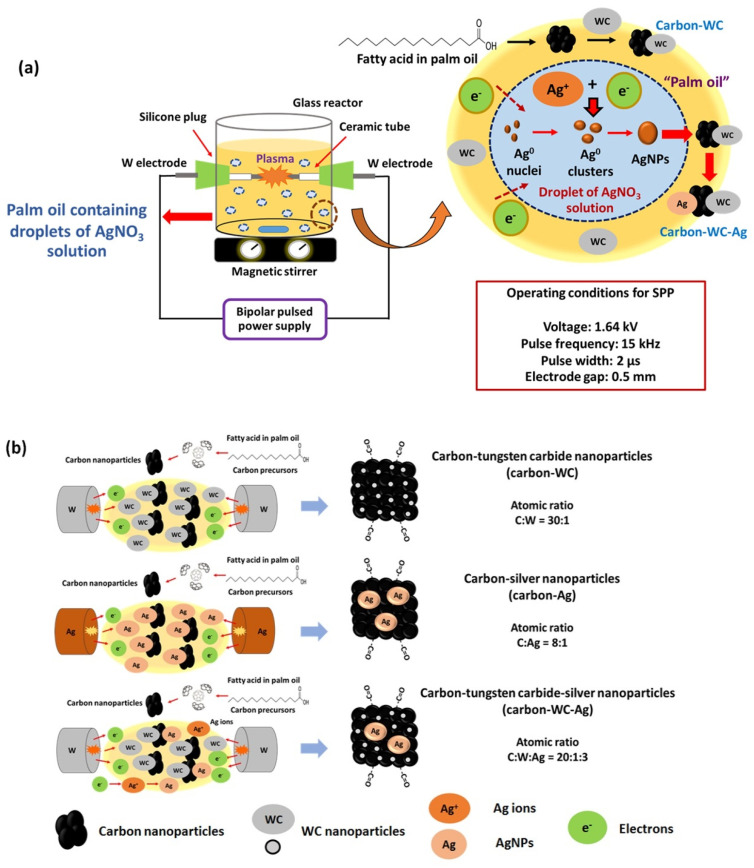
(**a**) Schematic diagrams of SPP and formation of AgNPs in a droplet of AgNO_3_ solution dispersed in palm oil, and (**b**) formation of carbon-based nanoparticles, i.e., carbon-WC, carbon-Ag and carbon-WC-Ag nanoparticles, synthesized by SPP using tungsten (W) and silver (Ag) electrodes.

The experimental setup of SPP is shown in Figure 1a. The plasma discharge was carried out at room temperature and atmospheric pressure by using a bipolar pulsed power supply (model Pekuris MPS-06K01C-WP1 from Kurita Seisakusho Co., Ltd., Kyoto, Japan). The plasma discharge was generated between a pair of metal electrodes equipped in a glass reactor that was filled with palm oil (40 mL). Two types of metal electrodes, i.e., tungsten (W) and silver (Ag) electrodes, were used to synthesize carbon-WC and carbon-Ag nanoparticles, respectively. The metal electrodes were insulated by ceramic tubes. The gap between the electrodes, voltage, pulse frequency, pulse width and plasma discharge time were 0.5 mm, 1.64 kV, 15 kHz, 2 µs and 90 min, respectively. Palm oil was used as a carbon precursor to synthesize carbon via SPP, whereas tungsten carbide nanoparticles (WCNPs) were generated by sputtering of tungsten electrodes and subsequently combining tungsten and carbon. For the synthesis of carbon-WC-Ag nanoparticles, AgNO_3_ solution (10 mL) at a concentration of 0.02 M was added into palm oil (40 mL) under strong agitation to form small droplets of AgNO_3_ solution that dispersed in palm oil while plasma discharge was taking place. The operating conditions for plasma discharge were the same as those for the synthesis of carbon-WC and carbon-Ag nanoparticles.

By applying SPP and using tungsten electrodes, palm oil and AgNO_3_ solution acted as carbon and Ag precursors to synthesize carbon-WC-Ag nanoparticles in one step. Based on the types of electrodes, i.e., tungsten and silver electrodes, there were three types of synthesized carbon-based nanoparticles, i.e., carbon-tungsten carbide (carbon-WC), carbon-silver (carbon-Ag) and carbon-tungsten carbide-silver (carbon-WC-Ag) nanoparticles, as illustrated in Figure 1b. After the plasma discharge, the synthesized carbon-based nanoparticles were collected and separated from palm oil by centrifugation at a rotational speed of 12,000 rpm for 15 min. Then, the synthesized carbon-based nanoparticles were rinsed with an excess amount of hexane to remove the residual oil, followed by centrifugation at a rotational speed of 12,000 rpm for 15 min. The washing process was repeated 3 times. The synthesized carbon-based nanoparticles were dried in an oven at 60 °C for 3 h.

### 2.3. Preparation of Neat PLA and PLA Composite Films

For preparation of neat PLA films, a PLA solution at a concentration of 4% (*w*/*v*) was prepared by dissolving PLA pellets of 4 g in chloroform 100 mL, followed by stirring at 60 °C for 2 h until PLA pellets were completely dissolved [60]. Then, the PLA solution was cast on Petri dishes and left at room temperature for 48 h to obtain neat PLA films. For preparation of PLA composite films, the fillers, i.e., activated charcoal, carbon-WC, carbon-Ag and carbon-WC-Ag nanoparticles, were dispersed in the PLA solutions, followed by sonication for 10 min. The filler contents in the PLA composite films were varied to be 0.25, 0.75, 1.23, 1.72 and 2.20 wt%, as shown in Table 1. After this, the PLA solutions containing each type of filler were cast on Petri dishes and left at room temperature for 48 h to evaporate the solvent, and PLA composite films having a thickness of 95.5 μm were obtained.

### 2.4. Characterization

The synthesized carbon-based nanoparticles were characterized in comparison with the commercial activated charcoal. Wide angle X-ray diffraction (WAXD) measurements were done by using SmartLab (Rigaku Corporation, Tokyo, Japan) with CuKα radiation (λ = 0.154 nm) operating at 40 kV and 30 mA. The values of degree of crystallinity (χ_c_) of neat PLA and the PLA composite films were determined according to Equation (1):

χ_c_ (%) = [*(A_c_*/*(A_c_ + A_a_)*] × 100% (1)

*A_c_* represents the area under the total crystalline region and *A_a_* represents the area under the total amorphous region [61].

Chemical compositions of activated charcoal and the synthesized carbon-based nanoparticles were analyzed by XPS (model Kratos Axis Ultra DLD from Kratos Analytical Co., Ltd., Manchester, UK) with an Al Kα X-ray source at 15 kV. Raman spectra of the synthesized carbon-based nanoparticles were detected by Spectra GX (Perkin Elmer Inc., Waltham, MA, USA). TEM and FE-SEM images of the synthesized carbon-based nanoparticles were taken using a JEOL (model JEM-2100 from JEOL Co., Ltd., Tokyo, Japan) and Hitachi (model s-4800 from Hitachi Co., Ltd., Tokyo, Japan), respectively. The elemental compositions of activated charcoal and the synthesized carbon-based nanoparticles were determined by scanning electron microscopy with energy dispersive X-ray (SEM-EDX) (Hitach Co., Ltd., Tokyo, Japan).

Surface and cross-sectional morphology of neat PLA and PLA composite films was observed by FE-SEM (Hitachi s-4800, Japan). All samples were coated with platinum before measurements. The thermal properties of neat PLA and PLA composite films were investigated using TGA (model Netzsch 209F3 from Netzsch Co., Ltd., Selb, Germany) under a nitrogen atmosphere. All samples were heated from 35 °C to 900 °C at a heating rate of 10 °C/min. The crystallization behavior of neat PLA and PLA composite films was investigated by differential scanning calorimetry (DSC) (model Netzsch 204F1 Phoenix from Netzsch Co., Ltd., Selb, Germany) under a nitrogen atmosphere. For all samples, the first heating scan was performed by heating the samples to 200 °C at a heating rate of 10 °C/min to eliminate thermal histories. After this, the samples were cooled down to 25 °C at a cooling rate of 10 °C/min. The second heating scan was performed by heating the samples to 200 °C at a heating rate of 10 °C/min. The glass transition temperature (T_g_), the cold crystallization temperature (T_cc_) and the melting temperature (T_m_) were determined from the second heating scan. Alumina crucible pans (Netzsch Co., Ltd., Selb, Germany) were used for TGA and DSC measurements. Young’s modulus and tensile strength of neat PLA and PLA composite films were measured by using a universal testing machine (model Lloyd LRX from Lloyd Instruments Co., Ltd., West Sussex, UK) according to the ASTM D882 with a 500-N load cell. Each datum was the average of 5 specimens. Antibacterial activities of neat PLA, PLA/activated charcoal, PLA/carbon-WC and PLA/carbon-WC-Ag composite films against *Escherichia coli* (*E. coli*) and *Staphylococcus aureus* (*S. aureus*) were investigated by using the colony-forming unit assay according to the modified procedure of Janpetch et al. 2016 [62]. The bacterial reduction rate (BRR) was calculated according to Equation (2):

Bacterial reduction rate = [*(N*_1_*− N*_2_*)/N*_1_] × 100% (2)

where *N*_1_ represents the number of colonies from the blank cell suspension and *N*_2_ represents the number of colonies from the cell suspension containing PLA samples.

### 2.5. Statistical Analysis

The significant differences in the selected parameters were evaluated via analysis of variance (ANOVA) according to Turkey’s HSD (honestly significant difference) test at *p*-value < 0.05 using IBM SPSS Statistics 26 software (SPSS Inc., Chicago, IL, USA).

## 3. Results and Discussion

### 3.1. Characterization of Carbon-WC and Carbon-WC-Ag Nanoparticles

According to the previous studies on SPP, active species such as H, C_2_ and W radicals were generated during plasma discharge in organic solvents by using tungsten electrodes due to the dissociation of the molecules of organic solvents and the sputtering of tungsten electrodes [26,33]. The formation of carbon mainly depended on the interactions of the generated C_2_ radicals with each other, resulting in the formation of polycyclic structures. In this study, the pathways of carbon formation from palm oil by SPP were postulated to involve the transformation of hydrocarbon moieties of fatty acids existing in palm oil to cyclic compounds. Subsequently, the cyclic compounds underwent recombination to form polycyclic structures and eventually converted to a network structure of carbon, as described in previous studies [33,36].

**Figure 2:**
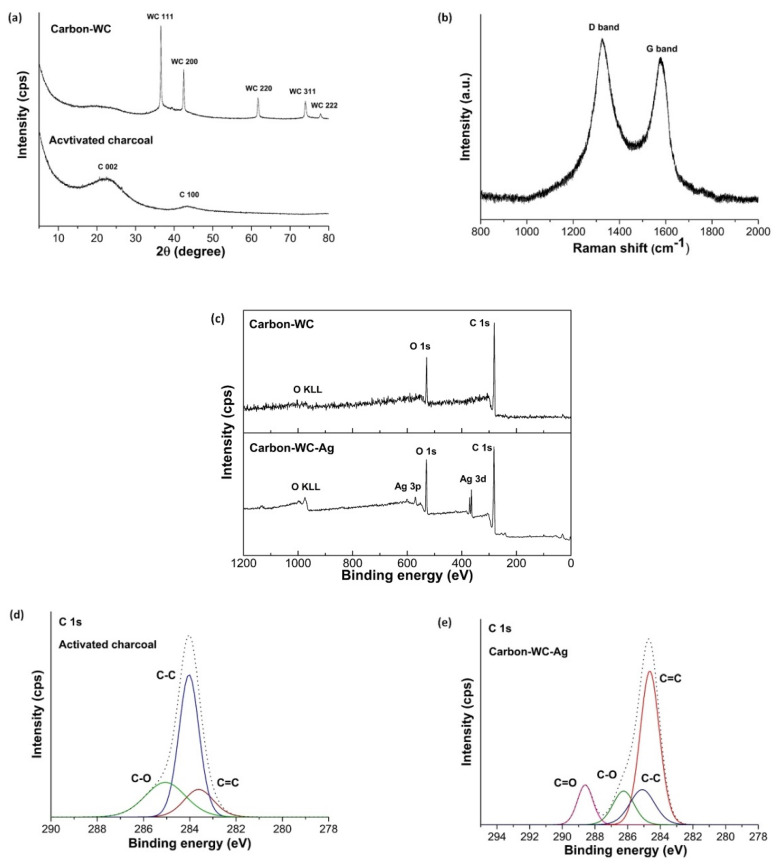
(**a**) XRD patterns of carbon-WC and activated charcoal, (**b**) Raman spectrum of carbon-WC, (**c**) wide-scan XPS spectra of carbon-WC and carbon-WC-Ag nanoparticles, and (**d**) narrow-scan XPS spectra of C 1s of activated charcoal and (**e**) carbon-WC-Ag nanoparticles.

In addition, tungsten carbide (WC) could be formed during plasma discharge by the reaction between W and C_2_ radicals and subsequently embedded in the simultaneously generated carbon, resulting in the formation of carbon containing tungsten carbide (carbon-WC). Figure 2a displays the XRD pattern of carbon-WC in comparison with that of activated charcoal. The characteristic peaks of activated charcoal at 2θ = 23° and 43° corresponding to the reflections of the (002) and (100) planes, respectively, indicated an amorphous state of activated charcoal. From the XRD pattern of carbon-WC, a very broad peak at 2θ = 25° suggested the amorphous state of carbon-WC. However, the peaks at 2θ = 36.7°, 42.6°, 61.8°, 74.1° and 78.0°, corresponding to the (111), (200), (220), (311) and (222) planes of metastable tungsten carbide (WC_1−x_), were also observed in the XRD pattern of carbon-WC [33,63]. Furthermore, a peak at 2θ = 38.5° corresponding to the (110) plane of tungsten metal appeared at a very low intensity. This might be explained by the fact that tungsten metal originated from the erosion of tungsten electrodes during plasma discharge [33,64]. The amount of tungsten metal incorporated into carbon-WC was relatively low due to the very low intensity of the peak of tungsten metal. Accordingly, carbon-WC synthesized by SPP in this study was referred to as the synthesized carbon that contained metastable tungsten carbide and a negligible amount of tungsten metal.

In Figure 2b, the Raman spectrum of carbon-WC consists of two peaks at 1350 cm^−1^ and 1580 cm^−1^ which correspond to the D band and G band, respectively. The D band is attributed to the occurrence of defects and disorder in the graphitic structure, whereas the G band is attributed to the formation of a well-organized graphitic structure. According to the obtained Raman spectrum, the intensity of the D band was slightly higher than the intensity of the G band, indicating an amorphous state of carbon-WC.

The wide-scan XPS spectra of carbon-WC and carbon-WC-Ag nanoparticles are shown in Figure 2c. The peaks of carbon C 1s and oxygen O 1s were detected for carbon-WC and carbon-WC-Ag nanoparticles, whereas the wide-scan XPS spectrum of carbon-WC-Ag nanoparticles shows the characteristic peaks of Ag 3d at 368.05 eV and 374.1 eV and the characteristic peaks of Ag 3p at 572.98 eV and 604.3 eV [20]. The narrow-scan XPS spectra of activated charcoal and carbon-WC-Ag nanoparticles are shown in Figure 2d,e, respectively. For activated charcoal, the C 1s spectra consisted of three peaks located at 283.70 eV, 284.00 eV and 285.3 eV, which corresponded to C=C, C-C and C-O, respectively. For carbon-WC-Ag nanoparticles, the C 1s spectra consisted of four peaks located at 284.64 eV, 285.09 eV, 286.26 eV and 288.59 eV, which corresponded to C=C, C-C, C-O and C=O, respectively. The C=O was present in carbon-WC-Ag nanoparticles but was absent in activated charcoal. It was suggested that carbonyl groups could be incorporated into the structure of carbon prepared by SPP [27,65].

**Figure 3:**
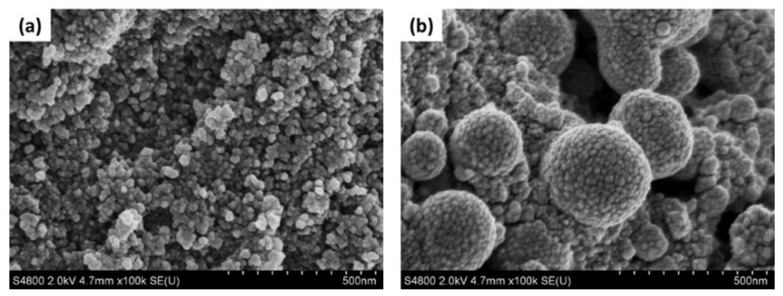
FE-SEM images of (**a**) carbon-WC and (**b**) carbon-WC-Ag nanoparticles at magnification of 100,000×.

**Figure 4:**
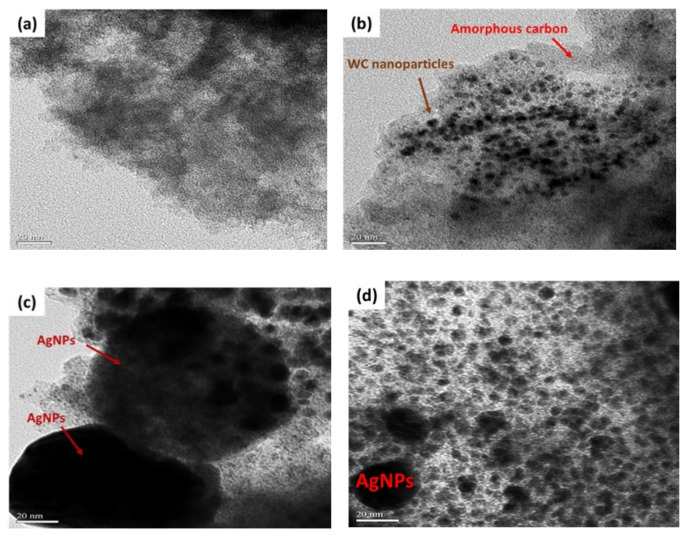
TEM images of (**a**) activated charcoal, (**b**) carbon-WC, (**c**,**d**) carbon-WC-Ag nanoparticles at magnification of 200,000×.

FE-SEM images of carbon-WC and carbon-WC-Ag nanoparticles are shown in Figure 3a,b, respectively. It was found that the particle sizes of carbon-WC and carbon-WC-Ag nanoparticles were in the ranges of 10–50 nm and 20–400 nm, respectively. Additionally, the average particle sizes of carbon-WC and carbon-WC-Ag nanoparticles were 22.60 nm and 129.06 nm, respectively. The particle size distribution of carbon-WC and carbon-WC-Ag nanoparticles is shown in Figure S1a,b. Interestingly, the FE-SEM image of carbon-WC-Ag nanoparticles (Figure 3b) shows spherical nanoparticles with bumpy surfaces. The presence of WC nanoparticles (WCNPs) in carbon-WC and carbon-WC-Ag nanoparticles was evidenced by TEM images, as shown in Figure 4b–d. It can be seen that WCNPs with particle sizes in the range of 2–5 nm were dispersed in amorphous carbon, whereas the TEM image of activated charcoal (Figure 4a) confirmed the amorphous state of activated charcoal. In Figure 4c,d, TEM images of carbon-WC-Ag nanoparticles show that AgNPs having particle sizes in the range of 10–400 nm and an average particle size of 72.74 nm were embedded in amorphous carbon, where WCNPs also dispersed. The particle size distribution of AgNPs existing in carbon-WC-Ag nanoparticles is shown in Figure S1c. The results of TEM are in good agreement with the results of XRD and Raman spectroscopy.

Furthermore, atomic percentages of each element in activated charcoal, carbon-WC, carbon-Ag and carbon-WC-Ag nanoparticles were investigated by scanning electron microscopy with energy dispersive X-ray (SEM-EDX) analysis and the results are shown in Table 2. The tungsten contents in carbon-WC and carbon-WC-Ag nanoparticles were 3.59% and 3.81%, respectively, whereas the silver contents in carbon-Ag and carbon-WC-Ag nanoparticles were 10.81% and 11.20%, respectively.

### 3.2. Morphology of Neat PLA and PLA Composite Films

The SEM images of the surface morphology of neat PLA and PLA/carbon-WC-Ag composite films with different filler contents of 0.25, 0.75, 1.23, 1.72 and 2.20 wt% were taken (Figure S2). While the neat PLA film (Figure S2a) had a smooth surface, the PLA/carbon-WC-Ag composite films (Figure S2b–f) had rugged surfaces due to the presence of carbon-WC-Ag nanoparticles in the composite films. The SEM images of the composite films indicated that, at the filler contents of 0.25, 0.75 and 1.23 wt% (Figure S2b–d), carbon-WC-Ag nanoparticles had low aggregation and could maintain random dispersion in the PLA matrix. However, at the higher filler contents of 1.72 and 2.20 wt% (Figure S2e–f), the aggregation of carbon-WC-Ag nanoparticles in the PLA matrix was obviously observed. Accordingly, the PLA/carbon-WC-Ag composite films with the filler content of 1.23 wt% were used for further studies. In addition, an SEM image of the cross-sectional morphology of the PLA/carbon-WC-Ag composite film at the filler content of 1.23 wt% was taken (Figure S3). It is clearly shown that carbon-WC-Ag nanoparticles were embedded in the PLA matrix and the average film thickness was measured to be 95.5 ± 0.52 μm (*n* = 3).

### 3.3. Thermogravimetric Analysis

TGA of neat PLA and PLA composite films, i.e., PLA/activated charcoal, PLA/carbon-WC, PLA/carbon-Ag and PLA/carbon-WC-Ag, at the filler content of 1.23 wt%, was performed and the values of the initial degradation temperature (T_int_), the 50% weight loss temperature (T_50_) and the degradation temperature (T_max_) are listed in Table 3. According to the TGA results, the T_int_ of PLA/carbon-WC-Ag composite film was significantly higher than that of the neat PLA film, indicating the increment of thermal stability of the PLA/carbon-WC-Ag composite film at the initial state of thermal degradation. This might be due to the heat capacity of WCNPs and AgNPs, which could absorb heat during the initial state of thermal degradation. It is known that various types of transition metals and alkali earth metal oxides favor the thermal decomposition of PLA, leading to a reduction in the thermal stability of PLA [7,66,67,68]. Compared with the neat PLA film, a reduction in T_50_ and T_max_ of PLA/carbon-WC composite films was observed. This result suggested that the presence of carbon-WC in the composite films could accelerate the thermal degradation of PLA. On the contrary, only a slight reduction in thermal stability of PLA/carbon-Ag composite film was observed when compared with the neat PLA film. Similarly, it has been reported that the presence of silver in the PLA matrix slightly affected the thermal stability of PLA composites [69,70].

Considering the T_50_ and T_max_ of PLA/carbon-WC-Ag composite films, the reduction in the thermal stability of PLA/carbon-WC-Ag composite films was less than that of PLA/carbon-WC composite films but higher than that of PLA/carbon-Ag composite films. As mentioned previously, carbon-WC could accelerate the thermal degradation of PLA, whereas AgNPs slightly affected the thermal stability of the composite films. It might be implied that the coexistence of AgNPs in carbon-WC-Ag could hinder the effect of WCNPs on the degradation of PLA upon heating. Nevertheless, the PLA/carbon-WC-Ag composite films had intrinsic thermal degradation properties. Therefore, the PLA/carbon-WC-Ag composite film is a potential candidate for use as an environmentally friendly material.

### 3.4. Differential Scanning Calorimetry

**Figure 5:**
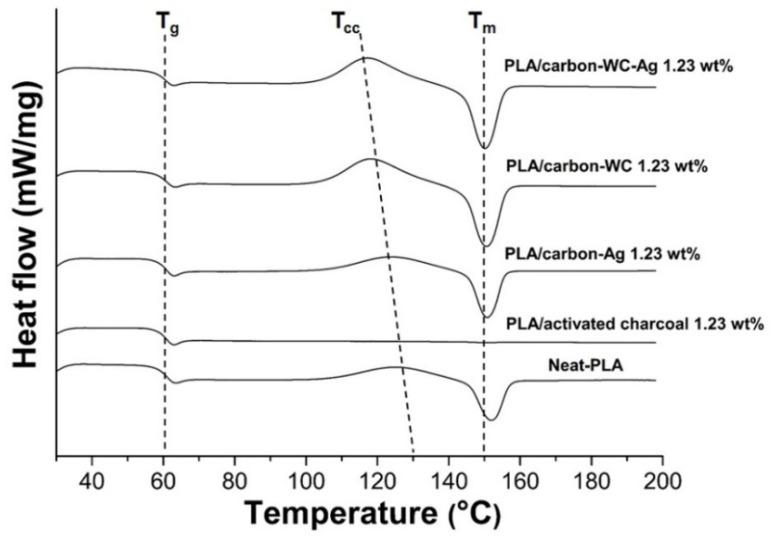
DSC thermograms of neat PLA and the PLA composite films at a filler content of 1.23 wt%.

The effect of the incorporation of the fillers, i.e., activated carbon, carbon-WC, carbon-Ag and carbon-WC-Ag nanoparticles, into PLA on the non-isothermal crystallization behavior of PLA was investigated by DSC via heating–cooling–heating scans and the results are shown in Figure 5. The glass transition temperature (T_g_), the cold crystallization temperature (T_cc_) and the melting temperature (T_m_) of neat PLA and the PLA composite films are detailed in Table 4. No significant change in the T_g_ of all the PLA composite films was observed when compared with the neat PLA film, indicating that the presence of the synthesized carbon-based nanoparticles, i.e., carbon-WC, carbon-Ag and carbon-WC-Ag, in PLA matrix did not affect the mobility of PLA chains in amorphous regions [39,71].

On the other hand, it was found that the incorporation of carbon-WC, carbon-Ag and carbon-WC-Ag nanoparticles as fillers in the PLA composite films resulted in shifts in the T_cc_ of the PLA composite films to lower temperatures compared with that of the neat PLA film, indicating the nucleating effect of the fillers. Due to the presence of polar functional groups such as carbonyl groups and high surface area, the synthesized carbon-based nanoparticles could interact with PLA and create nucleating sites to initiate the crystallization of PLA, resulting in an increase in the crystallization of PLA.

The presence of WCNPs and AgNPs in the synthesized carbon-based nanoparticles affected the nucleation ability of the synthesized carbon-based nanoparticles on the crystallization of PLA. It was found that the cold crystallization peak of PLA/carbon-WC was sharper and shifted to a lower temperature when compared to that of PLA/carbon-Ag (Figure 5). The lower T_cc_ in the second heating scan indicated the faster crystallization of PLA, which was induced by the added carbon-WC [10]. It might be implied that the presence of WCNPs in the synthesized carbon-based nanoparticles could promote the interaction between the synthesized carbon-based nanoparticles and PLA chains and subsequently enhance the nucleation ability of the synthesized carbon-based nanoparticles to accelerate the crystallization process of PLA.

Moreover, the T_cc_ of PLA/carbon-WC-Ag was lower than those of PLA/carbon-WC and PLA/carbon-Ag, suggesting the synergetic effect of the nucleation ability of WCNPs and AgNPs, accelerating the crystallization process of PLA. The T_m_ of all the PLA composite films shifted to lower temperatures when compared with that of the neat PLA film. This might be due to the transformation of the crystalline structure of PLA to the imperfect crystal forms during cold crystallization and the imperfect crystal forms could re-melt at a lower temperature [9,72].

### 3.5. X-ray Diffraction

**Figure 6:**
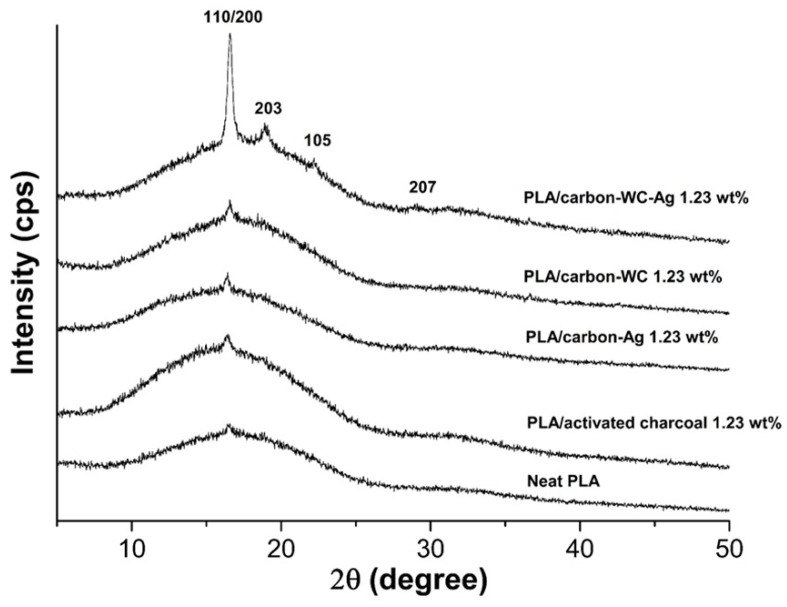
XRD patterns of neat PLA and the PLA composite films at a filler content of 1.23 wt%.

Figure 6 shows XRD patterns of neat PLA and the PLA composite films, i.e., PLA/activated charcoal, PLA/carbon-Ag, PLA/carbon-WC and PLA/carbon-WC-Ag, at the filler content of 1.23%wt. The XRD pattern of the neat PLA film exhibited a diffraction peak at 2θ = 16.64°, which corresponds to the (110)/(200) planes of crystalline PLA [73]. It was found that the intensity of the diffraction peak at 2θ = 16.64° in the XRD pattern of PLA/carbon-WC-Ag was much higher than those of neat PLA, PLA/activated charcoal, PLA/carbon-Ag and PLA/carbon-WC. From XRD analysis, it was found that the values of the degree of crystallinity (χ_c_) of neat PLA, PLA/activated charcoal, PLA/carbon-Ag, PLA/carbon-WC and PLA/carbon-WC-AgNPs were 1.43%, 1.52%, 2.20%, 5.94% and 14.23%, respectively. This evidence confirmed that carbon-WC-Ag nanoparticles could lead to an improved crystalline structure of PLA by the co-nucleation ability of WCNPs and AgNPs coexisting in carbon-WC-Ag nanoparticles.

### 3.6. Mechanical Test

In general, there are two possible factors that can influence the mechanical properties of PLA. The first is the dispersion of fillers in the PLA matrix and the second is the crystallinity of PLA, which depends largely on the crystallization behavior of PLA [39,74]. In this study, the Young’s modulus of the neat PLA film was 6.45 GPA. At the filler content of 1.23 wt%, the values of the Young’s modulus of PLA/activated charcoal, PLA/carbon-WC, PLA/carbon-Ag and PLA/carbon-WC-Ag composite films were 7.24, 7.74, 7.97 and 8.07 GPa, respectively. These results indicated that the presence of activated charcoal, carbon-WC, carbon-Ag and carbon-WC-Ag in the PLA matrix led to an increase in the Young’s modulus of PLA. The increased Young’s modulus was related to the DSC results showing that the co-nucleation of WCNPs and AgNPs coexisting in carbon-WC-Ag nanoparticles could accelerate the crystallization process of PLA, resulting in a better crystalline structure and higher Young’s modulus of PLA/carbon-WC-Ag composite films.

The values of tensile strength of the neat PLA, PLA/activated charcoal, PLA/carbon-WC, PLA/carbon-Ag and PLA/carbon-WC-Ag composite films were 19.92, 26.75, 21.16, 31.02 and 29.10 MPa, respectively. Due to the presence of some degree of aggregation of carbon-WC in the PLA matrix, the tensile strength of the PLA/carbon-WC composite film was not much higher than that of the neat PLA film. However, the incorporation of 1.23 wt% carbon-WC-Ag nanoparticles into the PLA matrix resulted in an increase in the tensile strength of PLA/carbon-WC-Ag composite films by 46.08% when compared with that of the neat PLA film. Since carbon-WC-Ag nanoparticles had bumpy surfaces, as evidenced by the SEM images, the surface roughness of carbon-WC-Ag nanoparticles might help to reduce the aggregation of carbon-WC-Ag nanoparticles in the PLA matrix, leading to a decrease in the stress concentrated point and consequently an increase in tensile strength [75]. Accordingly, it might be concluded that the incorporation of carbon-WC-Ag nanoparticles as fillers in the PLA matrix, even at a low filler content of 1.23 wt%, could significantly improve the Young’s modulus and tensile strength of the resulting PLA/carbon-WC-Ag composite films.

### 3.7. Antibacterial Test

Antibacterial activities against *E. coli* and *S. aureus* of the neat PLA and the PLA composite films were evaluated by colony-forming unit assay. The cell suspensions without the addition of tested samples were used as controls. It was found that the neat PLA film had no antibacterial activity against *E. coli* and *S. Aureus*, whereas the presence of activated charcoal and carbon-WC in the PLA composite films resulted in negligible inhibitory effects on *E. coli* and *S. Aureus*. The bacterial reduction rate (BRR) of the PLA composite film containing 1.23 wt% of carbon-WC-Ag nanoparticles against *E. coli* and *S. aureus* was 61.22% and 52.17%, respectively. It is well-known that AgNPs are used as a powerful antimicrobial agent. However, the deposition of AgNPs on a supporting material such as carbon may hinder the diffusion of AgNPs, resulting in lower antimicrobial activity than the corresponding unbound AgNPs. On the other hand, this may lead to better biocompatibility [22,76,77] when AgNPs deposited on a supporting material are incorporated into biomaterials for use in contact with living cells, such as in medical implants. Accordingly, the PLA/carbon-WC-Ag composites may be further developed for not only packaging materials but also biomedical applications.

## 4. Conclusions

Carbon-WC-Ag nanoparticles were simply synthesized in one step with the aid of SPP by using palm oil containing droplets of AgNO_3_ solution as carbon and silver precursors, respectively. Instead of using chemical agents, highly active species generated during plasma discharge induced the formation of carbon-WC-Ag nanoparticles. The synthesized carbon-WC-Ag nanoparticles were subsequently examined as multifunctional fillers for the preparation of PLA composite films fabricated by solution casting. The optimum content of carbon-WC-Ag nanoparticles in the PLA/carbon-WC-Ag composite film was 1.23 wt%. Interestingly, WCNPs and AgNPs coexisting in carbon-WC-Ag nanoparticles exhibited a co-nucleation ability to accelerate the crystallization process of PLA, resulting in an enhanced crystalline structure of the PLA/carbon-WC-Ag composite films. It was found that the degree of crystallinity of PLA/carbon-WC-Ag composite films was roughly ten times higher than that of neat PLA film. Consequently, the Young’s modulus and tensile strength of the PLA/carbon-WC-Ag composite films could be significantly improved. Moreover, WCNPs in the PLA composite films might be involved in the enhancement of the thermal degradation of PLA, leading to an advantage in their degradation. Due to the presence of AgNPs, the PLA/carbon-WC-Ag composite films exhibited antibacterial activities against *E. coli* and *S. aureus*. The evidence in this study indicates that SPP is a powerful tool for the synthesis of multicomponent nanoparticles. Moreover, multicomponent nanoparticles such as carbon-WC-Ag nanoparticles are useful as multifunctional fillers for the development of PLA composites aimed at packaging and biomaterial applications.

## Figures and Tables

**Table 1:** The amounts of fillers, PLA and chloroform for the preparation of PLA composite films.

Fillers (g)	PLA (g)	Chloroform (mL)	Filler Content in PLA Composite Films (wt%)
0.01	4.00	100.00	0.25
0.03	4.00	100.00	0.75
0.05	4.00	100.00	1.23
0.07	4.00	100.00	1.72
0.09	4.00	100.00	2.20

**Table 2:** Elemental compositions of activated charcoal, carbon-WC, carbon-Ag and carbon-WC-Ag nanoparticles (*n* = 3).

Type of Elements	Atomic Percentage (%) ± SD			
	Activated Charcoal	Carbon-WC	Carbon-Ag	Carbon-WC-Ag
Carbon (C)	94.42 ± 0.16	91.15 ± 0.46	82.10 ± 1.44	75.35 ± 2.04
Oxygen (O)	5.58 ± 0.16	5.35 ± 0.72	7.08 ± 0.85	9.64 ± 1.60
Tungsten (W)	-	3.59 ± 1.07	-	3.81 ± 0.83
Silver (Ag)	-	-	10.81 ± 1.06	11.20 ± 1.18

**Table 3:** Initial degradation temperature (T_int_), 50% weight loss temperature (T_50_) and degradation temperature (T_max_) of neat PLA and PLA composite films at a filler content of 1.23 wt%.

Composite Films	T_int_ (°C)	T_50_ (°C)	T_max_ (°C)
Neat PLA	102.4 ^a^	360.7 ^a^	366.5 ^a^
PLA/activated charcoal	104.9 ^a^	358.9 ^a,b^	364.8 ^a,b^
PLA/carbon-WC	107.1 ^a^	355.8 ^a^	363.3 ^a^
PLA/carbon-Ag	111.0 ^a^	359.5 ^a,b^	365.7 ^a^
PLA/carbon-WC-Ag	112.1 ^a^	357.8 ^a^	364.6 ^a,b^

The superscript letter “a” refers to significant differences among T_int_, T_50_ and T_max_ of the PLA composite films at *p*-value < 0.05. The superscript letter “b” refers to non-significant difference between T_50_ of PLA/activated charcoal and PLA/carbon-Ag and non-significant difference between T_max_ of PLA/activated charcoal and PLA/carbon-WC-Ag at *p*-value < 0.05.

**Table 4:** Glass transition temperature (T_g_), cold crystallization temperature (T_cc_) and melting temperature (T_m_) at the second heating scan of neat PLA and the PLA composite films at a filler content of 1.23 wt%.

Composite Films	T_g_ (°C)	T_cc_ (°C)	T_m_ (°C)
Neat PLA	60.1 ^b^	124.8 ^a^	152.0 ^a^
PLA/activated charcoal	59.6 ^b^	-	150.7 ^b^
PLA/carbon-WC	59.9 ^b^	118.1 ^a^	150.6 ^b^
PLA/carbon-Ag	59.8 ^b^	123.9 ^a^	150.7 ^b^
PLA/carbon-WC-Ag	59.3 ^b^	117.1 ^a^	150.1 ^b^

The superscript letter “a” refers to significant differences among T_cc_ of the PLA composite films at *p*-value < 0.05. The superscript letter “b” refers to non-significant differences among T_g_ and T_m_ of the PLA composite films at *p*-value < 0.05.

## Data Availability

The data presented in this study are available on request from the corresponding author.

## Supplementary Materials

The following are available online at https://www.mdpi.com/2073-4360/13/7/991/s1, Figure S1: Particle size distribution of (a) carbon-WC nanoparticles from SEM images, (b) carbon-WC-Ag nanoparticles from SEM images and (c) AgNPs existing in carbon-WC-Ag nanoparticles from TEM images (n = 300), Figure S2: SEM images of surface morphology of (a) neat PLA film and PLA/carbon-WC-Ag composite films with different filler contents of (b) 0.25, (c) 0.75, (d) 1.23, (e) 1.72 and (f) 2.20 wt%, Figure S3: FE-SEM image of cross-sectional morphology of PLA/carbon-WC-Ag composite films at the filler content of 1.23 wt% (magnification of 1000×).
