# Supplementary material for: Plasma-Assisted Synthesis of Multicomponent Nanoparticles Containing Carbon, Tungsten Carbide and Silver as Multifunctional Filler for Polylactic Acid Composite Films
Source: Polymers (Basel). 2021 Mar 24;13(7):991. doi: 10.3390/polym13070991 (PMC8037156; doi:10.3390/polym13070991)
Supplement: Supplementary file 1 [file polymers-13-00991-s001.pdf]

polymers

MDPI

Supplementary Materials

Plasma-assisted Synthesis of Multicomponent Nanoparticles containing Carbon, Tungsten Carbide and Silver as Multifunctional Filler for Polylactic Acid Composite Films

Nichapat Boonyeun[1,2], Ratana Rujiravanit[1,2,*] and Nagahiro Saito[3]

[1] The Petroleum and Petrochemical College, Chulalongkorn University, Bangkok10330, Thailand; Ratana.r@chula.ac.th, Nichapat.B@student.chula.ac.th
[2] Center of Excellence on Petrochemical and Materials Technology, Chulalongkorn University, Bangkok 10330, Thailand; Ratana.r@chula.ac.th, Nichapat.B@student.chula.ac.th
[3] Department of Chemical Systems Engineering, Graduate School of Engineering, Nagoya University, Nagoya 464-8603, Japan; Hiro@sp.material.nagoya-u.ac.jp
* Correspondence: Ratana.r@chula.ac.th; Tel.: 662 218-4132

3. Results and Discussion

Particle size distribution of carbon-WC, carbon-WC-Ag nanoparticles and AgNPs existing in carbon-WC-Ag nanoparticles

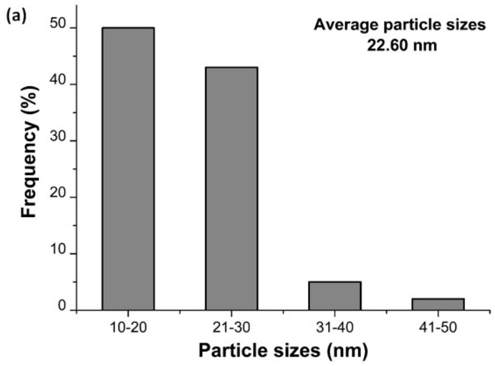

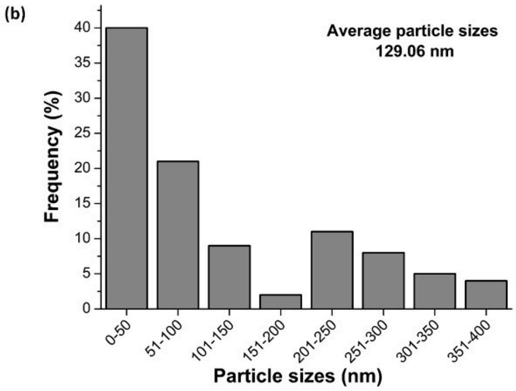

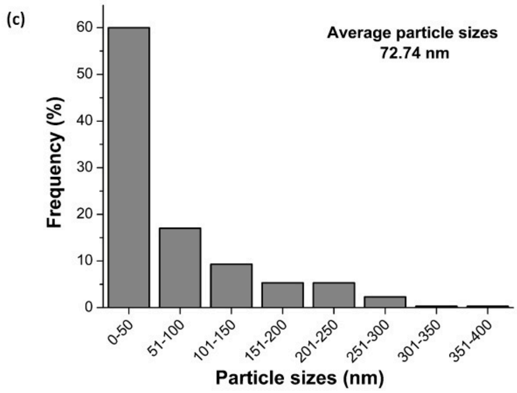

Figure S1. Particle size distribution of (a) carbon-WC nanoparticles from SEM images, (b) carbon-WC-Ag nanoparticles from SEM images and (c) AgNPs existing in carbon-WC-Ag nanoparticles from TEM images (n=300).

Surface morphology of neat PLA and PLA composite films having different filler contents

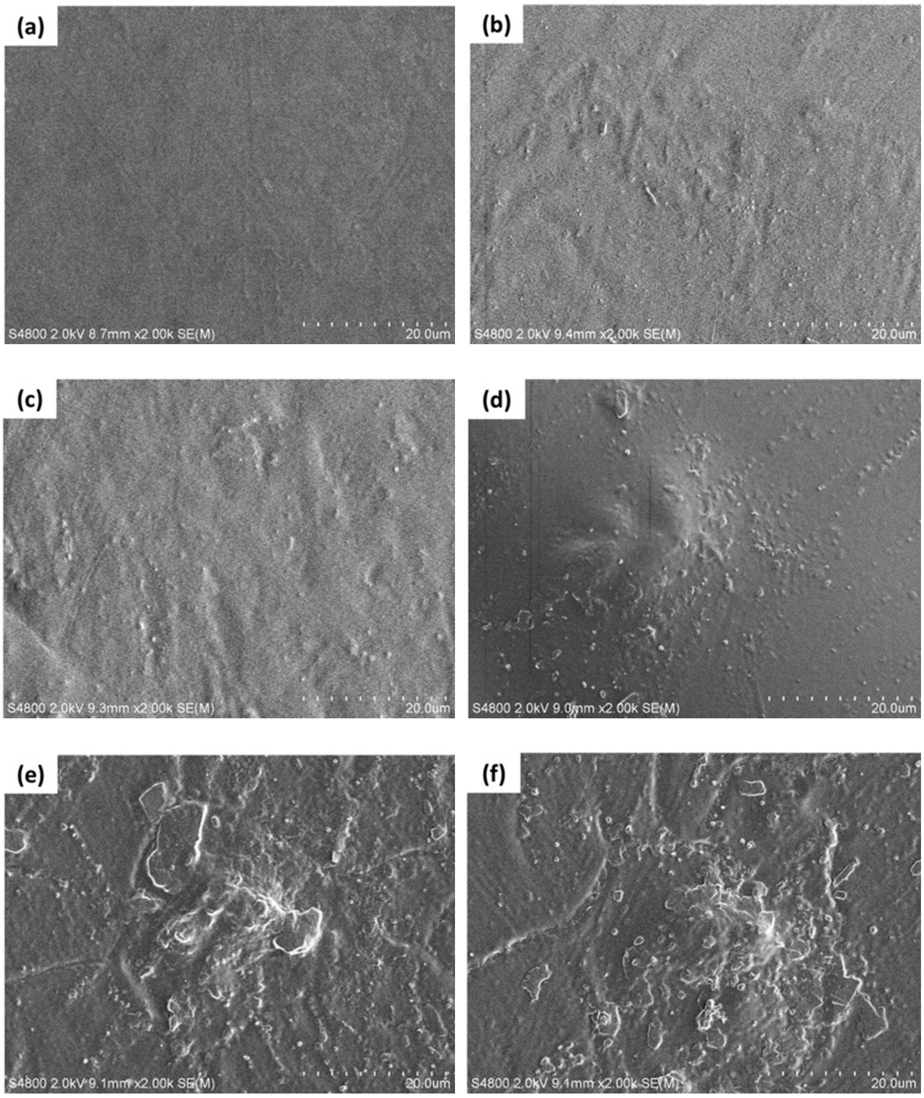

Figure S2. SEM images of surface morphology of (a) neat PLA film and PLA/carbon-WC-Ag composite films with different filler contents of (b) 0.25, (c) 0.75, (d) 1.23, (e) 1.72 and (f) 2.20 wt%.

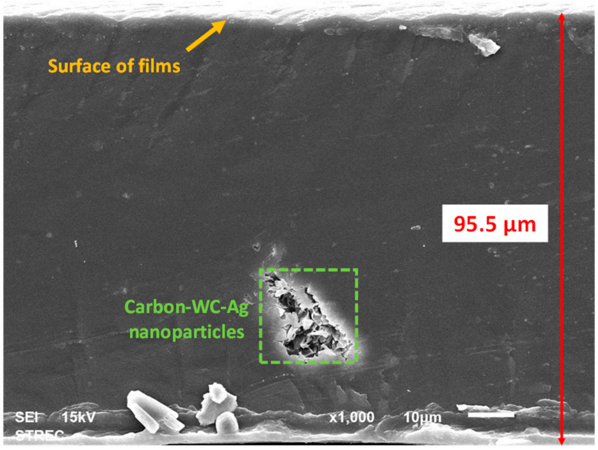

Figure S3. FE-SEM image of cross-sectional morphology of PLA/carbon-WC-Ag composite film at the filler content of 1.23 wt% (magnification of 1,000×).
